# Golden Years and Companion Animals: Investigating How the Human–Animal Bond Shapes Pet Wellness in Later Life from the Owner’s Perception

**DOI:** 10.3390/vetsci12080713

**Published:** 2025-07-29

**Authors:** Amira A. Goma, Emily Kieson

**Affiliations:** 1 Department of Animal Husbandry and Animal Wealth Development, Faculty of Veterinary Medicine, Alexandria University, Alexandria 21944, Egypt; 2 Department of Research, Equine International, Boston, MA 02120, USA; ekieson@equineintl.org

**Keywords:** elderly, health, quality of life, pets’ ownership, owner demographics

## Abstract

Most research studies have investigated the impact of pet ownership on the mental and physical well-being of elderly populations, supporting the beneficial effect that pets have on their owners. However, few researchers focused on the well-being of both owner and pet. The present study aimed to explore the well-being of pets owned by elderly individuals using an owner assessment tool and the relationship between elderly characteristics and the pet’s health-related quality of life based on the owner’s assessment of their pet’s well-being. Sixty elderly pet owners who made regular visits to veterinary clinics were selected to complete an electronic questionnaire about their pet’s health-related quality of life. The results identified a high agreement percentage on positive indicators related to the pet’s well-being such as “My pet wants to play and My pet responds to my presence” in the happiness domain, “My pet has more good days than bad days” in mental status, “My pet moves normally” in physical status and “My pet keeps him/herself clean” in hygiene which also resulted in a positive relationship with elderly age. Marital status influenced their responses to “My pet responds to my presence and My pet is as active as he/she has been”. The results also support the use of the applied questionnaire to help identify variables that contribute to a pet’s health-related quality of life. The correlation matrix revealed statistically significant positive associations (*p* < 0.001) among positively phrased items across all domains, as well as among negatively phrased items. These consistent alignments between direct and between reversed items suggest directional coherence and help mitigate potential response bias. Furthermore, the replication of these patterns across multiple domains reinforces the interpretation that the instrument captures a unified construct of pet well-being, In conclusion, based on subjective evaluation of pet-owner relationships, the ownership of pets by elderly individuals could be mutually beneficial to both elderly owners and their pets.

## Simple Summary

The beneficial effects of pet ownership on elderly individuals have been well documented; however, limited research exists regarding its impact on both owner and pets’ health and well-being. This study aimed to assess the effects of pet ownership by elderly individuals on their pets’ welfare. A total of 60 elderly pet owners who regularly visited veterinary clinics completed an electronic questionnaire evaluating their pets’ health-related quality of life. Findings indicated that, from the owners’ perspective, both the elderly individuals and their pets experienced comparable benefits in terms of companionship and physical well-being. These results underscore the significance of human–animal interactions and their mutually advantageous impact on both elderly owners and their pets.

## 1. Introduction

The mutually beneficial bond between humans and animals can be influenced by various factors such as daily human–animal interactions and the caretaking of the pet by the owner. These emotional, psychological, and physical interactions between humans and animals are the main components of this unique bond [1]. Pet ownership among elderly individuals has been associated with increased opportunities for social interaction and a sense of independence [2]. This result has also been seen in pet-assisted therapy involving individuals suffering from mental illness which helps them engage in a more lively and functioning life [3]. With regards to physical health, pet ownership may also benefit older adults with hypertension by encouraging engagement in daily caregiving tasks that promote a sense of purpose and routine. These activities can contribute to perceived improvements in physical functioning and overall well-being, improving mobility, increasing activity, and improving a sense of independence [4]. However, there is limited information about the health of the pet in these conditions and the resulting impact of ownership and human interactions.

The term ‘Quality of life’ (QOL) for animals was originally stated by Bono [5] and takes into consideration all aspects of animal welfare, from the prevention of mistreatment to the improvement of living conditions. Thus, QOL is a state of comfort or discomfort representing a combination of physical and non-physical factors [6]. The non-physical factors include satisfaction, sense of control, social relationship, emotional discomfort, and management of stress [7]. The primary goal of the QOL assessment in pets is focusing on the pet’s well-being [8]. Therefore, using a tool to gauge the quality of life of pets could introduce a new dimension into evaluating the relationship between the human and their pet [9].

Some studies suggest that the quality of life of pets is directly related to the owner’s perception of their relationship with their pet and the mutual experiences they share. Hewijnen et al. [10] stated that a good quality of life of both owner and pet is related to satisfaction, in which pets fulfill their owner’s psychological needs for autonomy, competence, and relatedness [11]. In these conditions, the pet might also benefit from enjoying interactions with their owner. For example, the presence of a human caretaker lowered stress in pets facing novel environments [12] suggesting that the relationship between pet and owner was also beneficial for the pet. Fewer studies, however, looked at the pet’s perspective, i.e., ownership’s positive or negative effects on the pet’s quality of life [13]. Marinelli [14] looked at the role of owner characteristics on pet’s quality of life. This study noted that a pet’s quality of life depends on some of their owner’s characteristics and listed the diversity of owner-related factors such as marital status, length of ownership, and previous experience which might affect the pet-owner bond and the quality of life of pets of various species. In the case of owner characteristics that affect pet quality of life, Budge et al. [15] determined that owners with a greater capacity to physical engage with their pets were more attached to them. Siniscalchi et al. [16] reported a correlation between the owner’s attachment style and the strength of the owner-dog bond, as evidenced through behavioral analyses and controlled observations. In another study, Sable [17] stated that pet-owner attachment strength could moderate the stress-reducing benefits of human–animal interaction to both. Thus, the quality of the human-pet relationship, owner attitudes toward animals, and experiences shared with, or result from, the companionship of animals could affect pet behavior and well-being.

The aim of the current study is to explore the well-being of pets owned by elderly individuals using a subjective assessment tool completed by the owners. Specifically, the study examines how various sociodemographic characteristics of elderly owners such as marital status, education level, and household composition relate to their pets’ well-being and health-related quality of life. Unlike most existing studies, which primarily focus on the benefits of pet ownership for older adults, this study shifts the perspective to the pets themselves. It investigates how the owner’s background and caregiving context may influence the pet’s quality of life, offering a more balanced, bidirectional view of the human–animal bond. The hypothesis is that pets owned by elderly individuals may experience improved well-being that might be related to the companionship and care they receive, mirroring the benefits often observed in their owners. This approach aligns with the One Welfare concept, which highlights the mutual influence of human and animal well-being, where promoting wellness in one can enhance the other. As described by García Pinillos et al. [18], One Welfare provides a framework to the interrelationships between animal welfare, human well-being and the physical and social environment that could serve as a platform for integrated policy and practice. Additionally, sociodemographic factors such as the owner’s marital status, education level, and presence of other household companions might relate to the pet’s well-being and health-related quality of life. By applying this lens, the study contributes a novel perspective to the literature on aging and companion animals.

## 2. Materials and Methods

### 2.1. Participants and Approvals

Sixty elderly individuals from Alexandria, Egypt—each aged between 60 and 80 years and owning one pet species participated in this study. Together, they represented a total of 60 pets. All participants reported making regular monthly visits to private and/or government veterinary clinics to monitor their pets’ health. The research protocol was approved by Faculty of veterinary medicine, Alexandria University’s Institutional Animal Care and Use Committee (IRB NO: 00007555-FWA NO: 000181699; ALEXU-IACUC, 013-20221212). An approval was obtained from the private and governmental veterinary clinics to participate in collecting the necessary data. Participants consented to participate in the study following an appropriate explanation of the study by members of the research team. In cases where participants were illiterate, the consent form was read aloud by researchers in the participant’s preferred dialect. Verbal consent was then obtained in the presence of a witness, in accordance with ethical guidelines for research involving vulnerable populations. This ensured that all participants fully understood the study and their rights before agreeing to take part. Participants’ privacy and anonymity were maintained, and confidentiality of the collected data was assured. The desire to withdraw from the study at any time was respected.

### 2.2. Data Collection

Data were collected through an electronic questionnaire developed by the researcher (AG). The questionnaire was translated into Arabic using a forward and back translation process by bilingual experts to ensure semantic equivalence. Comprehension was verified through pilot testing with a small group of elderly participants, and minor revisions were made for clarity. The final version of the questionnaire was sent to participants through the WhatsApp application. Participants’ responses about their pet’s health within the last two months were received and analyzed. In the case of illiterate participants, trained assistants read all questionnaire items aloud in the participant’s preferred language or dialect, ensuring full comprehension. The assistants then documented the participants’ verbal responses directly into the online form on their behalf. This procedure allowed for ethical inclusion while maintaining response accuracy and participant autonomy. Given that the questionnaire was distributed via WhatsApp, we took several steps to promote engagement and comprehension, including using clear, concise language and providing brief instructions at the beginning of the survey. Participants were recruited from a population with a known interest in pet care, which we believe increased the likelihood of sincere and attentive responses. We did not include formal attention check questions due to the need to keep the survey brief and accessible for an elderly population. The electronic questionnaire, Appendix A (Table A1 and Table A2) includes the following categories:

#### 2.2.1. Participants Socio-Demographic Data

The participants’ socio-demographic data included: age (60 to less than 65; 65 to less than 70; 70 and more), gender (male; female), marital status (single; married; widow; divorced), living arrangements (alone, with partner, with partner and children’s, with children’s), educational level (Illiterate; read and write; primary; preparatory; secondary; university; postgraduate), and income level (adequate; inadequate). Some health information was also included such as the presence (yes; no) and type of any medical diseases (hypertension; diabetes; cardiovascular; kidney; liver; others). The species of owned pet (dog; cat; decorated birds (e.g., budgerigars, cockatiels, lovebirds and canaries); decorated fish (e.g., Siamese Fighting Fish; goldfish; guppies); turtles and others such as parrots, hamsters, rats, mice and rabbits) and duration of ownership (less than one year; one to less than 5 years; more than 5 years) were also collected.

#### 2.2.2. Pets’ Health-Related Quality of Life (PHRQOL) Scale

Pet’s health related quality of life assessment in the questionnaire was based on the world Health Organization Quality of Life (WHOQOL) [19] and Villalobos [20]. The questionnaire questions were developed depending on the initial health-related quality of life scale for pets (PHRQOL) created by Lavan [21].

Although there is not a widely recognized scale specifically tailored to each pet species, some measures of well-being are broadly applicable across species. However, the method of assessment may need to be adapted based on the typical ways each species communicates its physical and emotional state. Therefore, a dog and cat-based scale created by Lavan [21] was used in this study with assessment of its validation for application to other pet species according to the international Society for Quality-of-Life Research (ISOQOL) guidance on implementing patient-reported outcomes in clinical practice to companion animal veterinary practice. It stated that “if there is not one available published validated questionnaire, then it is reasonable to devise one appropriate to need, with reference to human and veterinary literature. Amalgamating elements from a variety of existing tools may also prove useful” [22]. To make the scale more applicable across different species, we focused on broadly observable indicators of well-being that are meaningful regardless of species such as signs of positive and negative emotions, sleep and rest patterns, pain or discomfort, energy levels, mobility, daily activities, and overall health and care, as outlined in the WHOQOL framework. Furthermore, by grouping observable signs that may co-occur or reflect similar underlying issues, we aimed to facilitate more accurate and confident responses based on the owner’s general perception of their pet’s condition. Additionally, this approach was designed to accommodate a range of pet species, ensuring that the questionnaire remained broadly applicable while minimizing the need for species-specific phrasing.

The scale contains 15 items/questions grouped into four domains (happiness, physical status, hygiene, and mental status) shown in Table 1. Each domain related item was scored on a 5-level Likert scale, in which disagree = 1, slightly disagree = 2, neutral = 3, slightly agree = 4, agree = 5. In PHRQOL items (Table 1) description for fancy fish behaviors and indicators meaning:

- Panting: frequent air-gulping at the surface, evident by rapid opercular movements.
- Sleep: Characterized by significantly reduced activity, with the fish remaining in a fixed position.
- Pain: Indicated by an absence of response to food or any external interaction.
- Clean Appearance: Denoted by a lustrous look, free from dirt and with all scales intact.
- Shakes: Identified by erratic swimming behavior.
- Skin irritation: has visible skin lesions, discoloration, or abnormal patches on its body or fins
- Rough looking: fish’s scales or fins appear dull, clamped, frayed, or uneven

### 2.3. Statistical Analysis

Descriptive statistics were performed to analyze distribution of participants’ responses to the pet’s health-related quality of life items. After getting the number/percentage of responses in each level of the Likert scale the items marked as “disagree” were combined to give a number/percentage that reflected the combined score of “Total Disagree”. The same was performed for the levels for the category “agree”. Before performing the principal component analysis, a Kaiser-Meyer-Olkin (KMO) test was performed to determine the sampling adequacy of data to principal component analysis. Two principal components analysis (PCA) were performed, one for pets’ health-related quality of life questionnaire items to test the construct validity and another for both participant sociodemographic characteristics and their responses to pets’ health-related quality of life items to test for association. Although PCA is not a latent factor model, it serves as a preliminary dimensionality assessment tool. By summarizing shared variance among items, PCA helps identify item groupings that may reflect underlying constructs. To identify the reliability (internal consistency) of the pet’s health related quality items in questionnaire, Cronbach’s Alpha was determined for both the positive and negative questions separately. Also, Spearman correlation analysis was performed as a preliminary check to ensure directional coherence and mitigate response bias through determining the presence of significant positive correlation between the positive questions and also the negative questions separately. PCA components were interpreted alongside internal consistency metrics and domain specific correlations to provide initial evidence for multidimensional validity of the scale.

Further, multiple linear regression analysis with dichotomous moderator interaction and Spearman correlation were performed to evaluate the relationship between participants’ socio-demographic characteristics and their responses to pets’ health-related quality of life domains questions. The Mood’ median test was performed to determine the impact of participants’ sociodemographic characteristics on their responses to pets’ health related quality of life questionnaire items. The value of *p* < 0.05 was considered significant at first but later it was lowered to *p* < 0.01 to avoid Type I error. SPSS software (statistical package for social science software, version 25) was used in the analysis.

## 3. Results

### 3.1. Distribution of the Owners’ Responses to the Pets’ Health-Related Quality of Life Questionnaire Items

As an overall, the number of elderly respondents to the questionnaire was 60 (39 females and 21 males) and owning different types of pets (16 dog, 12 cat, 14 fancy birds, 10 fancy fish, 6 turtles, 2 others). The distribution of the elderly responses on the Likert scale are shown in Table 2. The highest agreement percentage between participants was found to Q1 and Q2 in the happiness domain (60.00; 61.70%, respectively), Q4 in mental status (61.60%), Q8 physical status (61.60%) and Q13 in hygiene (51.70%). This was followed by Q3 happiness (50.00%), Q6 mental (48.30%) and Q10 physical (50.00%). This indicates that there was agreement for most of the positive questions in all domains.

Moreover, the highest disagreement percentage was to Q5 mental status (51.60%), Q9 and Q12 physical (53.30; 66.70%, respectively), and Q14, Q15 hygiene (70.00; 55.00%, respectively). However, questions Q7 and Q11 physical (50.00; 46.70%, respectively) have the least disagreement. This reflects the disagreement for most of the negative questions in all domains that could indicate a good well-being of the pets under elderly ownership from the perspective of the owners.

### 3.2. Impact of Owner’s Socio-Demographic Characteristics on Response to Items of Pet’s Health-Related Quality of Life Domains Items

The Mood median test showed no significant effect for gender, living arrangements, education, income and type of disease in response to the items of pet health-related quality of life. However, age had a significant effect on participant responses to Q1–3 and Q9, while marital status influenced their responses to Q2, Q5, Q10, Q14. Also, type of pet owned effect on owners’ responses to Q2 and duration of ownership on response to Q6.

When going into further details (Table 3) for the impact of age on the participant responses to items of pet’s health-related quality of life, a greater number of participants aged 60 to less than 65 (*n* = 28) indicated through their responses to Q1, Q2, Q3, and Q9 that their pets were motivated to play, were responsive to their presence, and appeared to enjoy life, while also tending to lie in one place throughout the day. This was followed by participants aged 65 to less than 70 (*n* = 23), who expressed similar perceptions. While “wants to play” and “enjoys life” may appear similar, they were included as distinct items to capture different aspects of the pet’s behavior, one reflecting physical engagement and the other reflecting overall emotional well-being. Although lying in one place all day long may suggest inactivity, it does not contradict the desire to play. Pets can be physically restful yet mentally alert, ready to engage when stimulated. Their playfulness often surfaces in bursts, not constant movement.

Furthermore, marital status appeared to influence pet-related perceptions. Married participants (*n* = 30) more frequently indicated through their responses to Q2, Q5, Q10, and Q14 that their pets were responsive and active, yet also displayed signs of dullness or depression, and issues such as odor or skin irritation. These patterns were reported more often than among single participants (*n* = 23).

Regarding type of pet, participants reported owning a variety of pets. Table 4. Pet type influenced response patterns, particularly to Q2, which reflects pet responsiveness to the owner’s presence. The highest frequencies were observed among dog owners (*n* = 16), followed by those owning fancy birds (*n* = 14), cats (*n* = 12), and fancy fish (*n* = 10). Ownership duration also impacted responses. Participants who had owned their pets for less than one year (*n* = 34) more frequently endorsed Q6, indicating that their pets tended to sleep more and were less alert. This demonstrated that the most owned pet by participants was dogs for a duration of less than one year.

### 3.3. Association Between Owner’s Socio-Demographic Characteristics and Items of Pet’s Health-Related Quality of Life Domains

The KMO value was 0.798 (~0.80) which means that the present data was adequate for principal component analysis. The association between 24 items (15 items of the PHRQOL and 9 sociodemographic characteristics) were investigated by principal component analysis. Seven components have an eigenvalue greater than 1, explaining 73.23% of total variance. After observing the components matrix, two components (4, 6) were removed because of having coefficient <0.6. After that, five components (1, 2, 3, 5, 7) were retained that contain 12 items with coefficients > 0.6, explaining 62.63% of total variance (Table 5). Component 1 contains related positive questions (7) of pet health related quality of life scale which is the main component (r^2^ = 38.19%). This identifies the association between most of the positive questions of the questionnaire. Gender and marital status were grouped in component 3, whereas duration of ownership, education and human companionship (“living arrangements”) comes in separate components (2, 7, 5, respectively). However, there is no clear association between demographic data of participants and their pet well-being.

Further investigation was made by multiple linear regression analysis with dichotomous moderator interaction, to show the relationship between pet well-being questionnaire items and participant sociodemographic data. The first dichotomous moderator variable was gender. The analysis shows a positive relation of participant responses to Q1 (Equation (1)) with gender and human companionship (“Living arrangements”) within the elderly pet-owner population. This could indicate that gender and the presence of other people living with the elderly might play a role in perceived pet happiness and playfulness from their owners’ perspective. Further, relation of participant responses to Q2 (Equation (2)) with age and social interactions (“Living arrangements”) within the elderly population. The result reflects that the owner’s advanced age could make pets more responsive to their presence, which could also be positively affected by owner’s gender and number of human companionships. On the other hand, Q6 responses (Equation (3)) are negatively related to human companionship (“Living arrangements”) and gender, indicating that the number of the owner’s companions may impact the amount of time the pet sleeps, making it more awake and happier. Similarly, Q9 responses were found to be positively related with owner physical health (“type of disease”) and gender (Equation (4)), this shows that the owner’s type of disease together with gender could negatively affect the pet’s physical condition. However, responses to Q10 were related negatively with elderly education level (Equation (5)), which suggests that the education of the elderly pet owner may influence the pet’s physical activity from their owner’s perspective.

Q1 = −10.383 (+5.990) + 7.497 (+3.540, *p* = 0.040) elderly gender + 1.742 (+0.771, *p* = 0.029) elderly living arrangements × elderly gender (r^2^ = 26.70%) (1)

Q2 = −7.912 (+5.628) + 1.856 (+0.875, *p* = 0.040) elder age + 1.562 (+0.724, *p* = 0.036) elderly living arrangements × elderly gender (r^2^ = 32.00%) (2)

Q6 = +8.502 (+6.421) − 1.665 (+0.826, *p* = 0.050) elderly living arrangements × elderly gender (r^2^ = 27.70%) (3)

Q9 = +1.750 (+5.083) − 1.432 (+0.654, *p* = 0.034) elderly living arrangements × elderly gender + 0.681 (+0.286, *p* = 0.022) elderly type of disease × elderly gender (r^2^ = 48.80%) (4)

Q10 = −5.328 (+5.627) − 1.341 (+0.625, *p* = 0.037) elderly education level (r^2^ = 36.70%) (5)

Moreover, the multiple linear regression with income (Table 6) as modulator shows that responses to Q11 were negatively related with elderly age, elderly human companionship (“Living arrangements”), elderly income level, combined marital status and income level, combined education level and income level and also combined type of pet and income level. On the other hand, these responses were positively related with elderly marital status, elderly education level, type of pet owned by elderly, combined age with elderly income level, and combined human companionship (“Living arrangements”) and income level. These findings suggest that factors such as the owner’s older age, presence of companionship, and adequate income level may be associated with better perceived pet health, as reflected in lower reported frequencies of panting, a behavior that can sometimes indicate stress in companion animals. However, it is important to note that panting can also be a normal physiological response to heat or physical activity and should not be interpreted as a definitive marker of stress. Additionally, changes in marital status, education level, type of pet owned, and shifts in the number of human companions or income level with age may influence pet well-being, potentially contributing to variations in behaviors such as panting.

### 3.4. Correlation Between Sociodemographic Characteristics of the Owner’s and Pet’s Health-Related Quality of Life (PHRQOL) Domains Items

Further investigation into the relationship between the owner’s sociodemographic data and the items of the pet’s health-related quality of life was performed by determining correlations presented in Appendix B (Table A3) after splitting the responses into two groups according to participants’ gender which is the main dichotomous modulator. In male participants, a positive correlation was seen between “living arrangements” human companionship, and response to Q1 and Q2 of happiness domain (*p* = 0.022; 0.026, respectively) and type of disease with response to Q9 (*p* = 0.006) in physical domain. Whereas a negative relation was recorded between human companionship (“Living arrangements”), and response to Q6 in mental status (*p* = 0.029) and Q9 in physical status (*p* = 0.034). This shows that pets owned by male elderly owners who live with other people could be more attached to their owners and might have better mental and physical health according to owner’s perspective to their pet’s health. However, the type of disease from which the pet owner suffers could affect the pet’s physical condition.

With regards to the female elderly owners, a positive correlation was seen between age and response to Q2 and Q3 in the happiness domain (*p* = 0.005; 0.009, respectively); Q4 in mental status (*p* = 0.007) and Q8 (*p* = 0.046) in physical domain. However, age was negatively related with response to Q5 in mental (*p* = 0.009), Q7 and Q9 in physical (*p* = 0.013; *p* = 0.011) and Q15 in hygiene (*p* = 0.045) domain. Therefore, the pet owners who were identified as female reported that their advanced in age could have a positive impact on their perceived pet’s quality of life.

Further, elderly respondents who were identified as female showed that marital status was positively related to response to Q4 in the mental (*p* = 0.029) domain and Q10 in physical status (*p* = 0.010), whereas marital status was negatively related to response to Q5 in the mental domain (*p* = 0.016), Q7, Q9 and Q12 in physical status (*p* = 0.033; 0.006; 0.003, respectively), Q14 and Q15 in the hygiene (*p* = 0.030; 0.038, respectively) domain. This show that according to the perspective of female pet owners, marital status could positively affect their pet’s health related quality of life.

Furthermore, education was positively related to female response to Q9 in the physical domain (*p* = 0.018). Duration of ownership was positively related with response to Q7 in physical (*p* = 0.026) status while education was negatively related with response to Q10 in the physical (*p* = 0.005) domain. This suggests that, among female pet owners, the higher the education level and the longer the duration of pet ownership, could led to less attention given to the pet’s health.

### 3.5. Reliability and Construct Validity of Pets’ Health-Related Quality of Life (PHRQOL) Domains Items

The Cronbach’s Alpha for testing the internal consistency of the pet’s health related quality of life questionnaire items were 0.901 and 0.902, respectively, for the positive questions (*n* = 7) and negative ones (*n* = 8) which indicates an excellent reliability for the questionnaire applied. The KMO value was 0.890 (~0.90) which suggests that the present sample was adequate for principal component analysis. The association between the 15 items of the pet’s health related quality of life domains used in the questionnaire (7 positive and 8 negative) were examined by PCA (Table 7). Two components have an eigen value more than 1 explaining 66.32% of total variance. An observation of the two components matrix found that all items clustered in the first component explained 58.21% of total variance. The items were in a reverse association to each other as mentioned in the questionnaire, with the negative questions having a positive correlation coefficient of >0.60 whereas the positive questions have a negative correlation coefficient <0.60. This indicates the construct validity of the questionnaire items.

Further the Spearman correlation matrix (Appendix B Table A4) showed that there was a positive significant (*p* < 0.001) relationship between positive questions in all domains. Represented in Q1–3 of happiness, Q4 in mental status, Q8 and Q10 in physical status and Q13 in hygiene indicating that these questions could reflect the good pet’s health related quality of life. Moreover, there was also a positive relationship between the negative questions in all domains. Such as between Q5 and Q6 in mental status, Q7, Q9, Q11, Q12 in physical status and Q14 and Q15 in hygiene domain (*p* < 0.001), supporting the coherence of these questions and domains as indicator of pet’s health-related quality of life as all correlation coefficients (R-values) were higher than the tabled R-value which was 0.25.

Furthermore, there was a negative relationship between negative and positive questions (*p* < 0.001) in all domains. This can be seen between Q5 in mental condition and Q1–3 of happiness, Q4 mental, Q8 and Q10 physical and Q13 hygiene. See more in Appendix B Table A4. This inverse correlation pattern is consistent with theoretical expectations and supports the internal structure of the scale. The alignment between reversed and direct items reinforces that respondents interpreted the items coherently and in accordance with their intended directionality. Moreover, the consistency of these patterns across multiple domains strengthens the evidence that the instrument measures an integrated construct of pet well-being rather than isolated traits.

## 4. Discussion

Most previous studies had focused on the positive effect of pet ownership on elderly well-being, but there is limited information about how this ownership affects pets’ well-being, especially from the perspective of the owner. Therefore, the present study aimed to look at the well-being of pets owned by elderly and determine the relationships between owner characteristics and the pet’s health-related quality of life by using a questionnaire focusing on the subjective perspective of health and lifestyle of both owner and pet to assess the mutuality of the relationship. The hypothesis guiding this study is that pets owned by elderly individuals may experience enhanced well-being as a result of the companionship and care they receive which parallels the well-documented benefits that pets often provide to their owners. This perspective aligns with the One Welfare concept, which emphasizes the interconnectedness of human and animal well-being, suggesting that promoting health in one can positively influence the other. As outlined by García Pinillos et al. [18], One Welfare offers a framework for understanding the relationships between animal welfare, human well-being, and the broader physical and social environment, serving as a foundation for integrated policy and practice. Sociodemographic factors such as the owner’s marital status, education level, and the presence of other household members may also influence the pet’s well-being and health-related quality of life. By adopting this lens, the study offers a novel contribution to the literature on aging and companion animals and highlights the potential for developing targeted assessment tools to better evaluate pet well-being in diverse caregiving contexts.

The assessment and maintenance of the pet’s health-related quality of life (PHRQOL) is an essential topic when discussing pet ownership in domestic settings [21] or the role of pets as loving companions or status-enhancers [23]. This beneficial bond between human and animal has been influenced by various factors, such as the behaviors of humans and animals, animals’ health status, and owner characteristics [24] with research suggesting that the subjective satisfaction of the pet-owner relationship reflects the strength of this attachment [25]. People with high pet-attachment scores were found to have few social networks and adverse life events [26] suggesting that pets can be considered as an emotional support for those firmly attached to their animals [27]. In Melson [28], reported that children with lack of human social support benefit from having animals from whom they can get their emotional support. Elderly pet owners differ in various aspects from younger generations, though, including financial, quality and quantity of social contacts, level of education, and mindsets that might affect the owner-pet relationship and the pet’s health-related quality of life [29].

The higher percentage of agreement and significance of the elderly owner’s responses to questions determining the pet’s health related quality of life in the current study indicates an overall subjective assessment of good well-being of pets from the respondents’ perspective. Furthermore, the most significant responses were from the younger participants between the ages of 60 and 65 who were married and owned dogs for less than one year. In these cases, the pet’s well-being is determined through the owner’s observations of the pet’s expression of behaviors indicating motivation to play, “active”, “responding to their owner’s presence”, “enjoying life but sometimes lies in one place all day long” and “seems dull or depressed, not alert” and “smells like urine or has skin irritation”. Although the owners’ observation of the pet’s activity and play could be a clear indicator of potential positive well-being, the ability to notice problematic behaviors such as “seems dull or depressed, not alert” or “smells like urine or has skin irritation “could also indicate that the owners are aware of potential problems in their animals’ welfare.

The results could reflect the effect of age and marital status on the response to questionnaire items and agree with Marinelli et al. [14], who deduced that most dogs that had a high level of well-being were positively affected by their emotional bond with owners. Additional scientific evidence indicates the benefit to animals from the human–animal interaction [30,31] ranging from physiological [32] to endocrinological [33] aspects, especially in those involved in close relationships with humans. Tuber et al. [12] also showed that human companionship might reduce the impact of novelty or stress on pets even more than companionship with the same species and results in overall improvement on pet health and well-being.

### 4.1. Supporting the Use of the PHRQOL Assessment Scale

A valid constructive PHRQOL assessment scale is essential to identify various domains that independently contribute to the PHRQOL [8]. The current study indicated a strong correlation between the 15 items distributed into four domains used to assess the pet’s health-related quality of life, similar to the findings of Lynch et al. [34]. This was further confirmed by the principal component analysis. This together with the high value of Cronbach alpha found, supports the validity, reliability, and internal consistency of the PHRQOL-15 item assessment and suggests that it could detect PHRQOL changes in several domains in healthy pets, as a further support to the findings of Lavan [21]. The PHRQOL-15 emphasizes external and psychosocial parameters of QOL that are readily observable by an attentive pet owner. Therefore, this questionnaire could be a valuable tool for assessing the welfare and well-being of animals owned by elderly and used as a tool for guiding healthcare decisions. Hielm-Björkman et al. [35] used similar assessment items in the Finnish version of the Helsinki chronic pain index (HCPI) and reported that it provided a valid, reliable, and responsive tool for assessment of the quality of life in dogs with osteoarthritis. Piotti et al. [36] used the Milan Pet Quality of Life (MPQL) instrument, that captures four key domains of pet quality of life: physical, psychological, social, and environmental. They suggest that pets’ physical quality of life is largely influenced by their own demographics, life experiences, and personality traits. In contrast, the psychological quality of life of pets appears to be more closely associated with their owners’ demographics and personality. Consequently, according to Yeates and Main [37] a valid quality of life scale assessment for pets should have two main domains which are the mental state and external/physical domain. The first is the subjective and the latter objective. This is considered a balanced assessment. They also stated that the pet quality-of-life assessment is an important tool for small animals to reflect upon what is important for animals.

### 4.2. Impact of Additional Human Companions in the Home and Owner Physical Health

The study found that, when pets live in homes with additional humans (usually companions, family members, or spouses of the owner) or if the owner has health issues, these conditions could result in a significant change to the perceived mental and physical health of the animal. This could be especially true for pets living in homes where their primary owner had a permanent companion. In these circumstances, the owner may report improved positive mental and physical health or fewer reported behaviors that indicated poor mental and physical health of the animal. If the owner had poor physical health, however, they may have reported decreased pet welfare.

More specifically, according to factors related to others living in the same household, participant assessment of their pet’s poor mental/ physical condition by way of “sleeping more or lies in one place all day long” could have decreased, suggesting that the owner’s perception of sleep and mental health could also be affected by the number of individuals in the home. Further analysis found that the pet’s physical condition of “lying in one place all day long” may be positively affected by the participant’s having some type of physical disease which suggests that the owner’s perception of animal movement or actual animal movement could be affected by their own physical health and likely their own physical mobility.

There are few studies related to how marriage or human companionship within a household affects pet well-being. The Dog Aging Project study [38] suggests that households with married couples or multiple adults may include higher levels of income that can be used to improve pet health and well-being through better veterinary care but does not suggest a direct correlation between multiple adults and pet welfare.

### 4.3. Impact of Owner Age, Education, and Income

The findings of this study suggest that a combination of factors could influence the perception of owners to pet health, especially age, education, and household income. Higher levels of education were correlated with lower physical activity in pets, but higher ages and income in owners correlated with decreased reported stress behaviors in pets. Since the questionnaire relies on self-reported data from pet owners, the owner’s age may influence their perception of their pet’s well-being. This age factor may also affect the owner’s capacity to provide care, ultimately shaping the pet’s welfare and quality of life.

The results show that the pet’s physical activity could be affected by the participant education level, showing a decrease in pet physical activity with higher levels of education in their elderly owners. This may reflect lifestyle differences associated with educational attainments such as more sedentary routines, less time spent on outdoor activities, or differing perceptions of pet care needs. The findings differ in studies of younger demographics where increased education levels often lead to increases in physical activity and better nutrition for pets [38,39] suggesting that owner’s age may greatly impact the physical activity of pets despite higher education.

On another hand, reports of the pets “frequent panting” decreased with elderly aging, presence of other human companions, and an increase in income level. Furthermore, there was a difference in this with the change in marital condition together with the income level, the education level with the income, the type of disease with the income level and the type of pet owned according to elderly income level. Since panting is often considered a sign of stress in companion animals, the findings suggest that the pets may be less stressed under conditions where their primary owner is elderly or of higher socioeconomic status, has a higher income or education level, or where there is more than one person living in the house. These findings suggest that, when an owner is determining the health of their animal, there is a multi-layered impact of owner circumstances and home environmental conditions.

### 4.4. Influence of Gender Combined with Age and Additional Human Companions

As mentioned above, the beneficial bond between humans and animals is influenced by owner characteristics. In the present study, regression and correlation analyses were used to examine the relationship between owner characteristics and items within the pet’s health-related quality of life domains. The regression analysis showed that, for the happiness domain, there was a difference in perceived pets’ motivation to play with the owner or other members of the household and that this difference was based on the gender of the owner, suggesting that gender could either influence pet’s motivation or the perception of play in the pet. Since play can reflect both a pet’s desire for interaction and its need for physical activity, the gender of elderly pet owners may play a role in shaping the pet’s physical and mental well-being under these conditions. Additional regression findings show that the perceived pet’s response to the owner’s presence could be positively affected by the owner’s age and the presence of others (roommates or partners) that also lived in the environment. These findings suggest that older pet owners may contribute positively to their animals’ well-being, and that the presence of additional individuals in the household may also enhance the pet’s health and quality of life. These findings are supported by those found in the Dog Aging Project study [38] which also found that pets in household with married individuals showed higher welfare. The Dog Aging Project suggested that multiple incomes were likely responsible for the increased welfare which could also be the case in this study, but given the aging population, the correlation between multiple members, gender, and well-being of the animal might suggest that accessible companionship for the animal might also play a role.

With regards to gender, male pet owners with additional human household companions reported improved pet well-being. It was found that the reported level of attachment to other human companions correlated with their perceptions that their pets could also be more attached to these companions and enjoyed better mental and physical condition, suggesting that the perceived increase in companionship behaviors in the animals could also be related to overall perceptions of well-being in the pet. However, the findings from the study showed that the type of medical condition of the male participant negatively impacted the pet’s physical condition, suggesting the possibility of a strong connection between human physical health and perceived pet physical health.

With regards to female participants, aging factors of the participant positively affected their perception of their pet’s enjoyment of life, good mental health, hygienic, and physical condition, and subjective attachment to them. This finding suggests that the age of female pet owners may influence their perception of the pet’s well-being but may also impact how they interact with their animal which can directly affect the animal’s mental and physical health. Furthermore, the marital condition of female pet owners positively affected the mental, physical, and hygienic condition of pets, further suggesting that the mental and physical health of the owner impact the health of the pet. On the other hand, elderly female pet owners reported positive pet well-being if they had human companions or had physical ailments but reported lower pet well-being if they had higher education or had longer ownership of the pet suggesting that higher education or longer ownership might lead to neglect of the pet’s physical condition in these cases. Based on these findings, combinations of gender, histories and living conditions can impact on perceptions of pet well-being and therefore impact the likelihood of owners to support their pet’s well-being and physical health.

Overall, the findings indicate that the advanced of age of the owner could have a positive effect on pet’s well-being, especially for aged female owners. These findings are further supported by Pitteri et al. [29] who reported that human aging is associated with social and physical changes that influence the relationship characteristics with their pets. Bagley and Gonsman [40] also revealed a positive relationship between owner age and pet attachment level, who become more attached to their pets over time which was reflected in their pet’s quality of life.

Other studies support the outcomes related to gender differences. Although the study by Bagley and Gonsman [40] found a relationship between higher age and pet attachment, they also reported that the gender was not significantly related to the level of pet attachment. However, another study performed by Marinelli et al. [14] reported that dogs owned by men have the best physical condition.

In addition, there was a positive relationship between the marital status of the female owner and the pet’s health-related quality of life items, which was similarly reported by Marinelli et al. [14] who found a positive effect for the marital status on the level of pet’s care. On the other hand, in the current study, the level of education of owners may impact medical care in a different way where owners who did not graduate high school correlated with increases in the medical care level for their pets while those who graduated from high school resulted in decreases in medical care for pets. This is similar to the study of Marinelli et al. [14] in which they found a negative influence of the college education to pet care. These same findings were also reported by other authors [41,42].

### 4.5. Duration of Pet Ownership

The findings of the study suggest that longer durations of pet ownership may result in lower perceived pet well-being. It is possible that the duration of pet ownership may influence the relationship between the owner and the pet, but it is also possible that longer duration of pet ownership could mean higher age of the pet where the pet’s own health is in decline due to the pet’s advanced age. Similarly, Marinelli et al. [14] revealed that the duration of the relationship between human and pet negatively affects the pet quality of life, physical condition, and care in different ways, in which the physical condition decreased for those with a long association with their owner and also with the aging of the pet. It is possible that, with the increase in the length of the relationship between pet and owner, the owner’s attention to the pet’s needs may decrease. In this instance, however, the pet attachment to the owner becomes stronger. On another side, they deduced that those who shared a home with other people showed less passion and attachment to their pets. This may result from needing to split attention with other members of the household or from the pet forming closer bonds with other household members beyond the respondent. This contrasts with what is reported in the current study which is the positive effect of living with human companions. Since the current study focuses on the perceived well-being of the animal rather than a specific focus on the human owner, the findings may provide a more accurate representation of the pet’s experience within the household. These findings are supported by Adamelli et al. [43] who reported that cat quality of life and behavior are affected by the owner gender, education, previous experience and the number of family members, friends, and emotional bonds. Hoffman et al. [44] also found that owner level of attachment was positively related to their dog attention-seeking behavior in adults but not in children. This suggests that adults form an emotional bond with their dogs and seek out their attention. This helps to clarify the effects of owner demographic characteristics on their pet’s well-being and behavior. Further studies also elucidate the beneficial impact of pet and human relationship to both human and pets’ health and well-being [45,46,47,48,49,50,51], further supporting the findings of the study and that subjective assessment of pets by elderly pet owners can provide important insights into the health and well-being of their pets.

### 4.6. Limitations of the Study

The present study was limited to elderly individuals residing in a specific area of Alexandria, Egypt, who actively sought veterinary care for their pets and consented to participate. As such, the sample likely reflects a subset of pet owners with particular socioeconomic characteristics, namely, those with the financial means, physical mobility, and health literacy to access veterinary services. This introduces a potential selection bias, as it excludes elderly individuals who may own pets but lack the resources, access, or inclination to seek regular veterinary care. Consequently, the findings may not fully capture the experiences, challenges, or pet-owner dynamics of the broader elderly population, particularly those from underserved or rural communities. This limitation should be considered when interpreting the generalizability of the results to other settings or populations. Additional studies would need to be performed to determine if the findings in this study are representative of a larger population within Egypt or in other countries.

Further, the study did not investigate the actual well-being of the animal, but rather the owner’s perception of the animal’s well-being, which needs to be considered when interpreting the generalizability of the results to animal well-being. Moreover, the use of a WhatsApp-based questionnaire without formal attention check items, may affect the reliability of responses. Although the target population was likely to be engaged, future studies should include validity checks to ensure response accuracy in remote formats.

Also, pet age and health condition are another limitation as the pets in the current study are healthy young ones. While the PHRQOL scale was adapted from validated models, its application across diverse species, particularly non-mammalian pets such as fish and turtles, presents limitations. The scale relies on general behavioral indicators which may not fully capture species-specific expressions of well-being. Future research should focus on developing and validating species-specific PHRQOL tools to enhance accuracy and relevance.

## 5. Conclusions

The present study identified a higher percentage of agreement between elderly lifestyles to their perception of the positive well-being of their pets which could reflect the overall beneficial effect of elderly ownership on pets’ well-being from the owner’s perspective. More specific findings from the study emphasized the possible effects of age, gender, and household companions on the owner’s reported quality of life of their pet with the presence of additional household companions possibly having a significant impact on perceived pet well-being. These suggest that the complex relationship between elderly lifestyle and reported pet well-being can be mutually beneficial to both elderly and pets under the right conditions. Furthermore, the study supports the reliability and validity of the questionnaire for assessing the pet’s health-related quality of life. Therefore, the findings suggest that based on elderly pet owner’s perspectives of their pet’s quality of life and reported behavioral indicators of pet well-being, pets of elderly pet owners may benefit as much from the relationship as their owners.

## Figures and Tables

**Table 1:** Pet’s health-related quality of life (PHRQOL) domains items/questions that were translated into Arabic in the owner’s questionnaire.

Domain	Items/Questions
Happiness	(Q1) My pet wants to play
	(Q2) My pet responds to my presence
	(Q3) My pet enjoys life
Mental status	(Q4) My pet has more good days than bad days
	(Q5) My pet seems dull or depressed, not alert *
	(Q6) My pet sleeps more, is less awake *
Physical status	(Q7) My pet is in pain *
	(Q8) My pet moves normally
	(Q9) My pet lies in one place all day long *
	(Q10) My pet is as active as he/she has been
	(Q11) My pet pants frequently, even at rest *
	(Q12) My pet shakes or trembles occasionally *
Hygiene	(Q13) My pet keeps him/herself clean
	(Q14) My pet smells like urine or has skin irritation *
	(Q15) My pet’s hair is greasy, matted, rough looking *

* Indicate the reverse scored (negative) questions; Q (1–15): means question number.

**Table 2:** Distribution of the owner’s responses to the pet’s health related quality of life (PHRQOL) domains items on Likert scale.

Domain/Items	NO/%	Total Disagree	Total Neutral	Total Agree
**Happiness**				
(Q1) My pet wants to play	N	19	5	**36**
	%	31.70	8.30	**60.00**
(Q2) My pet responds to my presence	N	18	5	**37**
	%	30.00	8.30	**61.70**
(Q3) My pet enjoys life	N	18	12	**30**
	%	30.00	20.00	**50.00**
**Mental status**				
(Q4) My pet has more good days than bad days	N	15	8	**37**
	%	25.00	13.30	**61.60**
(Q5) My pet seems dull or depressed, not alert *	N	**31**	7	22
	%	**51.60**	11.70	36.70
(Q6) My pet sleeps more, is less awake *	N	27	4	**29**
	%	45.00	6.70	**48.30**
**Physical status**				
(Q7) My pet is in pain *	N	**30**	12	18
	%	**50.00**	20.00	30.00
(Q8) My pet moves normally	N	16	7	**37**
	%	26.70	11.70	**61.60**
(Q9) My pet lies in one place all day long *	N	**32**	8	20
	%	**53.30**	13.30	33.30
(Q10) My pet is as active as he/she has been	N	24	6	**30**
	%	40.00	10.00	**50.00**
(Q11) My pet pants frequently, even at rest *	N	**28**	6	26
	%	**46.70**	10.00	43.40
(Q12) My pet shakes or trembles occasionally *	N	**40**	4	16
	%	**66.70**	6.70	26.6
**Hygiene**				
(Q13) My pet keeps him/herself clean	N	11	6	**43**
	%	18.40	10.00	**51.70**
(Q14) My pet smells like urine or has skin irritation *	N	**42**	6	12
	%	**70.00**	10.00	20.00
(Q15) My pet’s hair is greasy, matted, rough looking *	N	**33**	6	21
	%	**55.00**	10.00	35.00

* Indicate the reverse scored (negative) questions.

**Table 3:** Impact of the Owner’s socio-demographic characteristics (age and marital status) on response to the pet’s health related quality of life (PHRQOL) domains items tested by Mood’s median test.

Items		Age			Marital Status			
		60 to Less than 65	65 to Less than 70	More than 70	Single	Married	Widow	Divorced
**Q1**	>Median	5	14	4				
	≤Median	23	9	5				
	Total	28	23	9				
	Median	4.00						
	*p*-value	0.007						
**Q2**	>Median	4	13	4	5	11	5	0
	≤Median	24	10	5	18	19	1	1
	Total	28	23	9	23	30	6	1
	Median	4.00			4.00			
	*p*-value	0.006			0.036			
**Q3**	>Median	9	16	5				
	≤Median	19	7	4				
	Total	28	23	9				
	Median	3.50						
	*p*-value	0.027						
**Q5**	>Median				16	12	0	1
	≤Median				7	18	6	0
	Total				23	30	6	1
	Median				2.00			
	*p*-value				0.009			
**Q9**	>Median	18	9	1				
	≤Median	10	14	8				
	Total	28	23	9				
	Median	2.00						
	*p*-value	0.014						
**Q10**	>Median				5	20	4	1
	≤Median				18	10	2	0
	Total				23	30	6	1
	Median				3.50			
	*p*-value				0.006			
**Q14**	>Median				15	14	0	0
	≤Median				8	16	6	1
	Total				23	30	6	1
	Median				1.00			
	*p*-value				0.027			

Represented in frequencies of participant responses to questionnaire questions according to their age and marital status distributed to being < or > than median, and total together with the median and significant level. Happiness: (Q1) My pet wants to play (Q2) My pet responds to my presence (Q3) My pet enjoys life; Mental status: (Q5) My pet seems dull’ or depressed, not alert; Physical status (Q9) My pet lies in one place all day long (Q10) My pet is as active as he/she has been; Hygiene: (Q14) My pet smells like urine or has skin irritation.

**Table 4:** Impact of the Owner’s socio-demographic characteristics (duration of ownership and type of pet owned) on response to the pet’s health related quality of life (PHRQOL) domains items tested by Mood’s median test.

Items		Duration of Ownership			Type of Pet Owned					
		Less than One Year	One to Less than 5 Years	More than 5 Years	Dog	Cat	Fancy Birds	Fancy Fish	Turtles	Others
**Q2**	>Median				4	9	3	2	2	1
	≤Median				12	3	11	8	4	1
	Total				16	12	14	10	6	2
	Median				4.00					
	*p*-value				0.043					
**Q6**	>Median	14	3	12						
	≤Median	20	7	4						
	Total	34	10	16						
	Median	3.00								
	*p*-value	0.037								

Represented in frequencies of participant responses to questionnaire questions according to the duration of ownership and type of pet owned distributed to being < or > than median, and total together with the median and significant level. Happiness: (Q2) My pet responds to my presence; Mental status: (Q6) My pet sleeps more, is less awake.

**Table 5:** Loading coefficients > 0.60 for 12 items (5 demographic) and (7 positive pet’s heath related quality of life) in 5 components generated by principal component analysis.

Items	Component				
	1	2	3	5	7
Gender			0.60		
Marital Status			0.70		
Living arrangements				0.66	
Education					0.63
Duration of ownership		0.74			
(Q1) My pet wants to play	0.79				
(Q2) My pet responds to my presence	0.84				
(Q3) My pet enjoys life	0.86				
(Q4) My pet has more good than bad days	0.68				
(Q8) My pet moves normally	0.79				
(Q10) My pet is as active as he/she has been	0.69				
(Q13) My pet keeps him/herself clean	0.70				
Proportion (%) of r^2^	38.19	7.50	6.98	5.46	4.51

Q1–3: happiness domain; Q4 mental status; Q8 and 10 physical status; Q13 hygiene domain.

**Table 6:** Relationship between the elderly responses to Q11 (My pet pants frequently, even at rest) in the pet well-being questionnaire items and participant sociodemographic data tested by multiple linear regression with income as modulator.

Q11	*R*^2^ = 22.60%
Constant	+17.824 (+7.277)
Elderly Age	−14.224 (+6.809, *p* = 0.043)
Elderly Marital status	+18.572 (+8.293, *p* = 0.030)
Elderly Living arrangements	−10.276 (+4.479, *p* = 0.027)
Elderly Education level	+5.236 (+2.020, *p* = 0.013)
Elderly Income level	−15.055 (+6.662, *p* = 0.029)
Type of Pet Owned	+6.151 (+2.553, *p* = 0.020)
Elderly Age × Elderly Income level	+13.903 (+6.831, *p* = 0.048)
Elderly Marital Status × Elderly Income level	−18.711 (+8.317, *p* = 0.030)
Elderly Living arrangements × Elderly Income level	+10.119(+4.493, *p* = 0.029)
Elderly Education level × Elderly Income level	−5.139 (+2.060, *p* = 0.016)
Elderly Type of Disease × Elderly Income level	−2.405 (+1.215, *p* = 0.054)
Type of Pet Owned × Elderly Income level	−6.079 (+2.572, *p* = 0.023)

**Table 7:** Loading coefficients for 15 items (8 negative) and (7 positive) pet’s heath related quality of life items generated by principal component analysis.

Items	Component
(Q1) My pet wants to play	−0.80
(Q2) My pet responds to my presence	−0.85
(Q3) My pet enjoys life	−0.85
(Q4) My pet has more good days than bad days	−0.68
(Q5) My pet seems dull or depressed, not alert *	0.80
(Q6) My pet sleeps more, is less awake *	0.64
(Q7) My pet is in pain *	0.83
(Q8) My pet moves normally	−0.80
(Q9) My pet lies in one place all day long *	0.70
(Q10) My pet is as active as he/she has been	−0.69
(Q11) My pet pants frequently, even at rest *	0.74
(Q12) My pet shakes or trembles occasionally *	0.71
(Q13) My pet keeps him/herself clean	−0.72
(Q14) My pet smells like urine or has skin irritation *	0.76
(Q15) My pet’s hair is greasy, matted, rough looking *	0.84
Proportion (%) of r^2^	58.21

* indicate the reverse scored (negative) questions.

## Data Availability

All data contained within this paper.

## Appendix A

**Table A1:** Socio-Demographic data collection.

Socio-Demographic Data							
Age	60 to less than 65		65 to less than 70			70 and more	
Sex	Male			Female			
Marital status	Single	Married		Widow		Divorced	
Living arrangements	Alone	With partner		With partner and children		With children	
Education level	Illiterate	Read and write	Primary	Preparatory	Secondary	University	Postgraduate
Income level	Adequate			Inadequate			
Disease	Yes			No			
Type of disease	Hypertension	Diabetes	Cardiovascular	Kidney		Liver	Others
Type of pet owned	Dog	Cat	Decorated birds	Decorated fish		Turtles	Others
Duration of pet ownership	Less than one year		One to less than 5 years			More than 5 years	

**Table A2:** Pet’s Health related quality of life (PHRQOL) items/questions. Each item was scored on a 5-level Likert scale, in which disagree = 1, slightly disagree = 2, neutral = 3, slightly agree = 4, agree = 5.

Items/Questions	1 = Disagree	2 = Slightly Disagree	3 = Neutral	4 = Slightly Agree	5 = Agree
(Q1) My pet wants to play					
(Q2) My pet responds to my presence					
(Q3) My pet enjoys life					
(Q4) My pet has more good days than bad days					
(Q5) My pet seems dull or depressed, not alert					
(Q6) My pet sleeps more, is less awake					
(Q7) My pet is in pain					
(Q8) My pet moves normally					
(Q9) My pet lies in one place all day long					
(Q10) My pet is as active as he/she has been					
(Q11) My pet pants frequently, even at rest					
(Q12) My pet shakes or trembles occasionally					
(Q13) My pet keeps him/herself clean					
(Q14) My pet smells like urine or has skin irritation					
(Q15) My pet’s hair is greasy, matted, rough looking					

Description for fancy fish behaviors and indicators meaning: Panting: Gulping oxygen at the surface, evident by rapid opercular movements; Sleep: Characterized by significantly reduced activity, with the fish remaining in a fixed position; Pain: Indicated by an absence of response to food or any external interaction; Clean Appearance: Denoted by a lustrous look, free from dirt and with all scales intact; Shakes: Identified by erratic swimming behavior; Skin irritation: has visible skin lesions, discoloration, or abnormal patches on its body or fins; Rough looking: fish’s scales or fins appear dull, clamped, frayed, or uneven.

## Appendix B

**Table A3:** Correlation matrix between owner’s socio-demographic characteristics and pet’s health related quality of life (PHRQOL) domains items represented in correlation coefficient and *p*-value.

Gender	Socio-Demographic Data	Happiness			Mental Status			Physical Status						Hygiene		
		Q1	Q2	Q3	Q4	Q5	Q6	Q7	Q8	Q9	Q10	Q11	Q12	Q13	Q14	Q15
**Male**	**Age**	0.258 0.265	0.283 0.218	0.262 0.260	0.019 0.935	−0.346 0.130	−0.087 0.705	−0.133 0.568	0.365 0.103	−0.343 0.128	0.027 0.908	−0.207 0.369	−0.096 0.676	0.022 0.926	−0.110 0.636	−0.149 0.519
	**Marital Status**	0.202 0.414	0.243 0.300	0.253 0.288	0.237 0.314	−0.188 0.425	0.048 0.865	−0.129 0.572	0.008 1.00	0.169 0.489	0.308 0.175	0.042 0.957	0.101 0.695	0.041 0.895	−0.138 0.564	−0.170 0.472
	**Living arrangements**	**0.505 *** **0.022**	**0.489 *** **0.026**	0.382 0.091	0.036 0.878	−0.125 0.596	**−0.483 *** **0.029**	−0.341 0.132	0.260 0.252	**−0.467 *** **0.034**	0.412 0.063	−0.257 0.262	−0.232 0.312	0.100 0.666	−0.068 0.771	−0.085 0.716
	**Education level**	0.204 0.371	0.146 0.524	0.031 0.896	0.171 0.455	−0.222 0.333	0.066 0.775	−0.036 0.880	0.181 0.422	0.218 0.336	−0.016 0.943	−0.142 0.551	−0.281 0.218	−0.102 0.656	0.054 0.809	−0.112 0.625
	**Type of disease**	−0.116 0.616	0.000 1.000	0.050 0.830	−0.046 0.837	−0.113 0.624	0.314 0.169	0.076 0.742	−0.152 0.503	**0.574 **** **0.006**	0.324 0.158	0.147 0.526	0.268 0.245	−0.229 0.320	0.061 0.793	0.023 0.916
	**Type of pet**	0.195 0.398	0.134 0.561	0.233 0.308	0.056 0.808	−0.102 0.651	−0.044 0.845	−0.098 0.666	0.147 0.524	−0.062 0.786	0.010 0.965	−0.312 0.168	−0.047 0.835	0.000 1.000	−0.086 0.712	−0.204 0.371
	**Duration of ownership**	−0.160 0.486	0.011 0.962	−0.156 0.499	−0.082 0.721	−0.020 0.933	0.160 0.490	0.043 0.850	−0.141 0.538	0.264 0.246	−0.027 0.908	0.391 0.076	0.133 0.566	−0.147 0.525	0.163 0.480	0.156 0.496
**Female**	**Age**	0.289 0.072	**0.444 **** **0.005**	**0.405 *** **0.009**	**0.418 **** **0.007**	**−0.421 **** **0.009**	−0.277 0.087	**−0.401 *** **0.013**	**0.324 *** **0.046**	**−0.405 *** **0.011**	0.243 0.134	−0.156 0.341	−0.241 0.142	0.003 0.985	−0.267 0.105	**−0.323 *** **0.045**
	**Marital Status**	0.219 0.181	0.237 0.147	0.302 0.064	**0.354 *** **0.029**	**−0.389 *** **0.016**	−0.214 0.192	**−0.346 *** **0.033**	0.178 0.276	**−0.441 **** **0.006**	**0.410 **** **0.010**	−0.178 0.274	**−0.464 **** **0.003**	−0.020 0.904	**−0.354 *** **0.030**	**−0.335 *** **0.038**
	**Living arrangements**	−0.019 0.914	0.122 0.454	−0.079 0.628	−0.147 0.371	−0.112 0.491	−0.037 0.823	0.036 0.830	−0.036 0.826	0.100 0.541	0.048 0.766	−0.072 0.654	0.231 0.157	0.158 0.333	0.160 0.333	−0.086 0.597
	**Education level**	−0.105 0.523	−0.145 0.379	−0.168 0.304	−0.259 0.114	0.304 0.060	0.301 0.063	0.257 0.115	−0.118 0.467	**0.379 *** **0.018**	**−0.438 **** **0.005**	0.187 0.256	0.150 0.364	−0.283 0.084	0.125 0.447	0.282 0.081
	**Type of disease**	0.166 0.312	0.068 0.678	0.062 0.701	0.176 0.282	0.053 0.748	−0.033 0.841	−0.122 0.450	−0.213 0.190	−0.077 0.638	0.104 0.528	−0.184 0.258	−0.078 0.624	−0.075 0.652	0.230 0.156	0.036 0.823
	**Type of pet**	0.021 0.902	−0.032 0.848	0.227 0.162	−0.175 0.289	−0.137 0.413	−0.144 0.383	−0.271 0.095	0.122 0.465	−0.013 0.939	0.145 0.382	0.197 0.230	0.034 0.836	0.125 0.446	−0.045 0.788	−0.074 0.657
	**Duration of ownership**	−0.072 0.658	−0.157 0.335	−0.181 0.263	−0.001 0.997	0.302 0.061	0.299 0.062	**0.359 *** **0.026**	−0.173 0.284	0.230 0.154	−0.202 0.211	0.040 0.806	0.136 0.404	−0.049 0.771	0.150 0.362	0.148 0.369

Happiness: (Q1) My pet wants to play (Q2) My pet responds to my presence (Q3) My pet enjoys life; Mental status: (Q4) My pet has more good days than bad days (Q5) My pet seems dull or depressed, not alert (Q6) My pet sleeps more, is less awake; Physical status: (Q7) My pet is in pain (Q8) My pet moves normally (Q9) My pet lies in one place all day long (Q10) My pet is as active as he/she has been (Q11) My pet pants frequently, even at rest (Q12) My pet shakes or trembles occasionally; Hygiene: (Q13) My pet keeps him/herself clean (Q14) My pet smells like urine or has skin irritation (Q15) My pet’s hair is greasy, matted, rough looking. * *p* < 0.05; ** *p* < 0.01.

**Table A4:** Correlation matrix between the pet’s health related quality of life (PHRQOL) domains items represented in correlation coefficient and *p*-value.

Domain /Items	Happiness			Mental Status			Physical Status						Hygiene		
	Q1	Q2	Q3	Q4	Q5	Q6	Q7	Q8	Q9	Q10	Q11	Q12	Q13	Q14	Q15
**Happiness**															
**Q1**		0.844 ** <0.001	0.725 ** <0.001	0.455 ** <0.001	−0.608 ** <0.001	−0.526 ** <0.001	−0.538 ** <0.001	0.637 ** <0.001	−0.579 ** <0.001	0.571 ** <0.001	−0.682 ** <0.001	−0.665 ** <0.001	0.445 ** 0.001	−0.392 ** 0.003	−0.646 ** <0.001
**Q2**	0.844 ** <0.001		0.782 ** <0.001	0.534 ** <0.001	−0.737 ** <0.001	−0.487 ** <0.001	−0.655 ** <0.001	0.698 ** <0.001	−0.516 ** <0.001	0.534 ** <0.001	−0.556 ** <0.001	−0.498 ** <0.001	0.503 ** <0.001	−0.530 ** <0.001	−0.649 ** <0.001
**Q3**	0.725 ** <0.001	0.782 ** <0.001		0.604 ** <0.001	−0.649 ** <0.001	−0.612 ** <0.001	−0.764 ** <0.001	0.658 ** <0.001	−0.526 ** <0.001	0.660 ** <0.001	−0.507 ** <0.001	−0.452 ** <0.001	0.524 ** <0.001	−0.553 ** <0.001	−0.649 ** <0.001
**Mental** **status**															
**Q4**	0.455 ** <0.001	0.534 ** <0.001	0.604 ** <0.001		−0.507 ** <0.001	−0.294 * 0.021	−0.570 ** <0.001	0.535 ** <0.001	−0.450 ** 0.001	0.488 ** <0.001	−0.409 ** 0.002	−0.470 ** <0.001	0.458 ** <0.001	−0.518 ** <0.001	−0.597 ** <0.001
**Q5**	−0.608 ** <0.001	−0.737 ** <0.001	−0.649 ** <0.001	−0.507 ** <0.001		0.532 ** <0.001	0.701 ** <0.001	−0.627 ** <0.001	0.422 ** 0.001	−0.550 ** <0.001	0.541 ** <0.001	0.431 ** 0.001	−0.526 ** <0.001	0.722 ** <0.001	0.784 ** <0.001
**Q6**	−0.526 ** <0.001	−0.487 ** <0.001	−0.612 ** <0.001	−0.294 * 0.021	0.532 ** <0.001		0.502 ** <0.001	−0.358 ** 0.006	0.461 ** 0.001	−0.534 ** <0.001	0.464 ** <0.001	0.450 ** <0.001	−0.387 ** 0.003	0.337 ** 0.010	0.410 ** 0.001
**Physical status**															
**Q7**	−0.538 ** <0.001	−0.655 ** <0.001	−0.764 ** <0.001	−0.570 ** <0.001	0.701 ** <0.001	0.502 ** <0.001		−0.642 ** <0.001	0.528 ** <0.001	−0.614 ** <0.001	0.503 ** <0.001	0.438 ** 0.001	−0.586 ** <0.001	0.627 ** <0.001	0.718 ** <0.001
**Q8**	0.637 ** <0.001	0.698 ** <0.001	0.658 ** <0.001	0.535 ** <0.001	−0.627 ** <0.001	−0.358 ** 0.006	−0.642 ** <0.001		−0.597 ** <0.001	0.441 ** 0.001	−0.531 ** <0.001	−0.605 ** <0.001	0.522 ** <0.001	−0.712 ** <0.001	−0.648 ** <0.001
**Q9**	−0.579 ** <0.001	−0.516 ** <0.001	−0.526 ** <0.001	−0.450 ** 0.001	0.422 ** 0.001	0.461 ** 0.001	0.528 ** <0.001	−0.597 ** <0.001		−0.411 ** 0.001	0.552 ** <0.001	0.614 ** <0.001	−0.454 ** 0.001	0.453 ** <0.001	0.525 ** <0.001
**Q10**	0.571 ** <0.001	0.534 ** <0.001	0.660 ** <0.001	0.488 ** <0.001	−0.550 ** <0.001	−0.534 ** <0.001	−0.614 ** <0.001	0.441 ** 0.001	−0.411 ** 0.001		−0.429 ** 0.001	−0.517 ** <0.001	0.356 ** 0.006	−0.366 ** 0.003	−0.559 ** <0.001
**Q11**	−0.682 ** <0.001	−0.556 ** <0.001	−0.507 ** <0.001	−0.409 ** 0.002	0.541 ** <0.001	0.464 ** <0.001	0.503 ** <0.001	−0.531 ** <0.001	0.552 ** <0.001	−0.429 ** 0.001		0.672 ** <0.001	−0.491 ** <0.001	0.471 ** <0.001	0.684 ** <0.001
**Q12**	−0.665 ** <0.001	−0.498 ** <0.001	−0.452 ** <0.001	−0.470 ** <0.001	0.431 ** 0.001	0.450 ** <0.001	0.438 ** 0.001	−0.605 ** <0.001	0.614 ** <0.001	−0.517 ** <0.001	0.672 ** <0.001		−0.379 ** 0.003	0.409 ** 0.001	0.509 ** <0.001
**Hygiene**															
**Q13**	0.445 ** 0.001	0.503 ** <0.001	0.524 ** <0.001	0.458 ** <0.001	−0.526 ** <0.001	−0.387 ** 0.003	−0.586 ** <0.001	0.522 ** <0.001	−0.454 ** 0.001	0.356 ** 0.006	−0.491 ** <0.001	−0.379 ** 0.003		−0.616 ** <0.001	−0.636 ** <0.001
**Q14**	−0.392 ** 0.003	−0.530 ** <0.001	−0.553 ** <0.001	−0.518 ** <0.001	0.722 ** <0.001	0.337 ** 0.010	0.627 ** <0.001	−0.712 ** <0.001	0.453 ** <0.001	−0.366 ** 0.003	0.471 ** <0.001	0.409 ** 0.001	−0.616 ** <0.001		0.712 ** <0.001
**Q15**	−0.646 ** <0.001	−0.649 ** <0.001	−0.649 ** <0.001	−0.597 ** <0.001	0.784 ** <0.001	0.410 ** 0.001	0.718 ** <0.001	−0.648 ** <0.001	0.525 ** <0.001	−0.559 ** <0.001	0.684 ** <0.001	0.509 ** <0.001	−0.636 ** <0.001	0.712 ** <0.001	

Happiness: (Q1) My pet wants to play (Q2) My pet responds to my presence (Q3) My pet enjoys life; Mental status: (Q4) My pet has more good days than bad days (Q5) My pet seems dull or depressed, not alert (Q6) My pet sleeps more, is less awake; Physical status: (Q7) My pet is in pain (Q8) My pet moves normally (Q9) My pet lies in one place all day long (Q10) My pet is as active as he/she has been (Q11) My pet pants frequently, even at rest (Q12) My pet shakes or trembles occasionally; Hygiene: (Q13) My pet keeps him/herself clean (Q14) My pet smells like urine or has skin irritation (Q15) My pet’s hair is greasy, matted, rough looking. * *p* < 0.05; ** *p* < 0.01.
